# Dry skin and the use of leave‐on products in nursing care: A prevalence study in nursing homes and hospitals

**DOI:** 10.1002/nop2.204

**Published:** 2018-09-27

**Authors:** Anna Lechner, Nils Lahmann, Andrea Lichterfeld‐Kottner, Ursula Müller‐Werdan, Ulrike Blume‐Peytavi, Jan Kottner

**Affiliations:** ^1^ Department of Dermatology and Allergy, Clinical Research Center for Hair and Skin Science Charité – Universitätsmedizin Berlin Berlin Germany; ^2^ Department of Geriatrics, Nursing Research Group in Geriatrics Charité – Universitätsmedizin Berlin Berlin Germany; ^3^ University Centre for Nursing and Midwifery Gent University Gent Belgium

**Keywords:** care, care activities, dermatology, hospital care, nursing home care, practice nursing, quality of care

## Abstract

**Aims:**

To describe the prevalence of dry skin in nursing homes and hospitals and to describe relationships between topical skincare interventions and dry skin.

**Design:**

Two multicentre descriptive cross‐sectional prevalence studies.

**Methods:**

The studies were performed in German nursing homes and hospitals in 2015 and 2016. Data were collected by trained nurses based on a standardized data collection form. The severity of dry skin was measured using the Overall Dry Skin Score.

**Results:**

In total, 1,662 nursing home residents and 1,486 hospital patients participated. The prevalence of dry skin was 41.2% in nursing homes and 55.2% in hospitals. In case of skincare dependency, the proportions of participants with dry skin were higher, particularly in hospitals (70.2%). In both institutions, the application of leave‐on products increased when dry skin was present but remained lower in hospitals. Considering the high amount of skin dryness in skincare‐dependent participants, interventions seem not to be successful. Results indicate a need for skincare improvement in future.

## 1 INTRODUCTION

Skin dryness (xerosis cutis) is the most common skin problem in aged populations (Kottner, Lichterfeld, & Blume‐Peytavi, 2013; White‐Chu & Reddy, 2011). Previous research showed that in German hospitals and nursing homes, almost every second person was affected by dry skin (Lechner, Lahmann, Neumann, Blume‐Peytavi, & Kottner, 2017; Lichterfeld, Lahmann, Blume‐Peytavi, & Kottner, 2016). Skin dryness can severely reduce the quality of life, and accompanied itching can disrupt sleep and causes emotional distress (Chang et al., 2017; Izumi et al., 2017). The disturbed skin barrier increases the risk of secondary infections (Chang et al., 2017), and studies indicate that xerosis may be an independent risk factor for the development of pressure ulcers/injuries (Coleman et al., 2014; Lechner et al., 2017). This emphasizes the importance of appropriate skincare interventions in nursing practice. For the treatment of dry skin, the use of non‐irritating skin cleansing procedures and the generous and frequent use of leave‐on products are recommended (Lichterfeld et al., 2015; Moncrieff et al., 2013). However, little is known about the actual skincare practice in nursing care (Kottner, Rahn, Blume‐Peytavi, & Lahmann, 2013). Results of a systematic review indicate that skin cleansing practice in nursing is currently rather based on “custom and practice” than on an evidence‐based approach (Cowdell & Steventon, 2015). This finding is supported by health service research results from German institutional long‐term and home care settings indicating that there is a huge unexplained heterogeneity in product selection and frequencies of applications (Kottner, Boronat, Blume‐Peytavi, Lahmann, & Suhr, 2014; Kottner, Rahn, et al., 2013; Rahn, Lahmann, Blume‐Peytavi, & Kottner, 2016). Whether specifically dry skin is appropriately treated with leave‐on products in daily nursing practice is unknown. Therefore, this study focuses on skincare‐dependent nursing home residents and hospital patients with dry skin who receive skincare interventions by nurses.

### 1.1 Background

The outermost keratinized skin layer, the stratum corneum, is mainly responsible for the level of skin hydration (Paul et al., 2011). Natural moisturizing factors in the corneocytes and intercellular lipids are crucial elements of the stratum corneum contributing to skin integrity by limiting transepidermal water loss (Moore & Rawlings, 2017; Paul et al., 2011). An imbalance in the composition of different components of the stratum corneum and changes in keratinization processes can lead to a disturbed skin barrier function and finally to skin dryness (White‐Chu & Reddy, 2011).

Clinical signs of skin dryness comprise scaling, roughness, redness and/or cracks (Serup, 1995). There are numerous factors which may cause skin dryness like endocrine disorders, genetic predisposition, climate or medication (Paul et al., 2011; White‐Chu & Reddy, 2011). Fluid intake is believed to affect the skin hydration as well, but the empirical evidence supporting this association is week (Akdeniz, Tomova‐Simitchieva, Dobos, Blume‐Peytavi, & Kottner, 2018), especially in care‐dependent aged adults (Akdeniz, Boeing, et al., 2018).

Especially aged people are vulnerable to xerosis due to structural and functional changes such as a decreased sebum and sweat production, elevated skin surface pH or declined cell replacement (Hodgkinson, Rhonda, & Wilson, 2006; Kottner, Lichterfeld, et al., 2013). The lower extremities were shown to be most often affected by dry skin in aged people (Lichterfeld et al., 2016; Lichterfeld‐Kottner, Lahmann, Blume‐Peytavi, Mueller‐Werdan, & Kottner, 2018; Smith, Atkinson, Tang, & Yamagata, 2002). In German nursing home residents as well as in hospital patients, a prevalence of dry skin of at least 40% was observed (Lechner et al., 2017; Lichterfeld et al., 2016). In a recent prevalence study in aged nursing home residents (65+ years), dermatologists diagnosed dry skin including mild forms in nearly every nursing home resident (Hahnel, Blume‐Peytavi, Trojahn, Dobos, Jahnke, et al., 2017; Hahnel, Blume‐Peytavi, Trojahn, Dobos, Stroux, et al., 2017).

Dry skin can be effectively treated with adequate skincare interventions (Chang et al., 2017; Hahnel, Blume‐Peytavi, Trojahn, Dobos, Stroux, et al., 2017; Hahnel, Blume‐Peytavi, Trojahn, Dobos, Jahnke, et al., 2017; Moncrieff et al., 2013). It is recommended to use lipophilic leave‐on products containing humectants such as urea, dexpanthenol or glycerine. The application should be performed at least twice daily or more often, depending on the severity of skin dryness (Guenther et al., 2012; Lichterfeld et al., 2015). Nurses play a key role in the quality of skincare (Kottner & Surber, 2016). An evidence‐based approach to assess the skin status and to promote skin health is important (Cowdell & Steventon, 2015; Kottner & Surber, 2016). Irrespectively of the large heterogeneity and uncertainty about skincare in nursing practice (Kottner & Surber, 2016; Kottner, Rahn, et al., 2013), there is no evidence, whether skincare provision is targeted to treat signs of dry skin.

## 2 THE STUDY

### 2.1 Aim

The aim of this study was to describe the prevalence of dry skin in nursing home residents and hospital patients and to describe relationships between the use of topical leave‐on products and dry skin in skincare‐dependent patients and nursing home residents.

### 2.2 Design

The current exploratory study is part of a primary research study performed in 2015 and 2016. Annual multicentre descriptive cross‐sectional prevalence studies are performed by the Department of Geriatrics at the Charité—Universitätsmedizin Berlin, and methods have been described previously (Kottner, Wilborn, Dassen, & Lahmann, 2009; Lahmann, Halfens, & Dassen, 2005). All hospitals and nursing homes in Germany were invited to participate. At a specific day, data collection was performed by trained nurses in all institutions based on a standardized data collection form.

### 2.3 Participants

Nursing home residents, as well as hospital patients in Germany, were invited to take part in the prevalence surveys. For inclusion, a minimum age of 16 and informed consent were required.

### 2.4 Data collection

Each participating institution appointed a local study coordinator who was trained by the investigators. For data collection, the coordinator trained the responsible nurses, who examined, assessed and interviewed the participating patients and residents. The standardized data collection form contained variables about demographics, health conditions, skin status and skincare activities.

Four skin areas were examined for the assessment of the occurrence and severity of skin dryness: face, trunk, hands and arms, as well as feet and legs. The severity of dry skin was measured using the Overall Dry Skin Score, which categorizes clinical signs of dryness from 0 (=absent) ‐ 4 (=large scales, roughness, redness, cracks/fissures). It is a clinical scoring system proposed by the European Group on Efficacy Measurement of Cosmetics and other Topical Products for dry skin assessment (Serup, 1995), and the validity was supported recently by Kang et al. (2014). The variable “dry skin overall” was defined as having dry skin (category 1 or higher) at the “face” and/or “trunk” and/or “hands and arms” and/or “feet and legs”. Category 1 was defined as mild dry skin, categories 2–4 as moderate‐to‐severe dry skin. The variable mobility was classified from 0 (=complete dependent) to 5 (=complete independent) according to the Care Dependency Scale (Dijkstra, Buist, Moorer, & Dassen, 2000). Pruritus was recorded when the participants scratched themselves or felt itching. The variable skincare dependency referred to participants who were not able to perform their skincare all by themselves. Skincare dependency was coded as a yes/no variable. Following skincare activities performed by the nursing staff were recorded: being partially or completely washed, showered, bathed and/or creamed. In addition, the body area was documented at which these skincare activities were performed. Leave‐on products were defined as cosmetic products which are intended to stay in prolonged contact with the skin such as creams or ointments (European Union, 2009). The completed data forms were sent back and analysed by the Department of Geriatrics at the Charité—Universitätsmedizin Berlin.

### 2.5 Ethical considerations

Study participation was voluntary. Before collecting data, informed consent was obtained by patients and residents personally or by a legal representative. The ethics commission of the Medical Association of Berlin approved this cross‐sectional survey (Eth‐837‐262/00).

### 2.6 Data analysis

A formal sample size calculation was not performed. Based on the previous annual sample sizes, the number of participants was expected to be sufficient for the descriptive analysis and group comparisons. In a first step, demographic and other characteristics of nursing home residents and hospital patients were described using proportions, means and spread parameters. The prevalence of “dry skin overall,” “pruritus,” “skincare dependency” and for the skincare activities “washing,” “showering,” “bathing” and “leave‐on products applied” was calculated as proportions of the total samples multiplied by 100. The 95% confidence intervals were calculated using the Wilson Score method.

In a next step, the proportions of dry skin in skincare‐dependent participants were described. Regarding the four body parts “face,” “trunk,” “arms and hands” and “legs and feet,” a differentiation between the dry skin levels “mild forms” (ODS = 1) and moderate‐to‐severe forms (ODS 2–4) was made. Chi‐square tests were applied to analyse whether there were statistical significant differences between nursing homes and hospitals regarding skincare‐dependent participants with dry skin.

The use of leave‐on products in skincare‐dependent participants was analysed in nursing home residents and hospital patients separately. Skincare‐dependent participants were allocated to the groups “without dry skin,” “with mild form” and “with moderate‐to‐severe form,” and the number and proportion of participants receiving leave‐on products by nurses was calculated. Differences between skin dryness levels and the application of leave‐on products were tested by performing chi‐square tests regarding each body site.

For all chi‐square tests, an alpha‐level of 0.05 (two‐sided) was considered as statistically significant.

### 2.7 Validity, reliability and rigour

Several studies supported the internal and external validity, reliability and rigour of the annually performed prevalence studies in Germany (Kottner et al., 2009; Lahmann et al., 2005). The study results obtained from the annually performed prevalence studies described above seem to be accurate and generalizable to the German hospital and nursing homes populations (Kottner et al., 2009; Lahmann et al., 2015). The design of this study was derived from the Dutch national registration form which was developed to measure the prevalence of pressure ulcers/injuries and was tested for reliability and feasibility by Bours, Halfens, Lubbers, and Haalboom (1999) which later became the international LPZ project (Nie‐Visser et al., 2013; Watson, 2013).

## 3 RESULTS

### 3.1 Participants

In 2015 and 2016, a total of 1,662 nursing home residents and 1,486 hospital patients participated. Characteristics of the study sample are shown in Table 1. The mean age was 81.0 years in nursing homes and 70.2 years in hospitals. The average body mass index was 26.0 kg/m^2^ in residents and 27.1 kg/m^2^ in hospital patients. The proportion of females was higher in nursing homes than in hospitals (65.8% vs. 49.5%).

**Table 1:** Demographic characteristics, signs of dry skin and skincare activities (2015 and 2016)

Demographics	Nursing homes (*N* = 1,662)	Hospitals (*N* = 1,486)
Age (years)		
Mean (*SD*)	81.0 (12.2)	70.2 (16.0)
Median (IQR)	84.0 (77.0–89.0)	75.0 (61.0–81.0)
BMI (kg/m^2^)		
Mean (*SD*)	26.0 (5.5)	27.1 (5.7)
Median (IQR)	25.4 (22.3–28.7)	26.3 (23.3–29.9)
Female (*N*, %)	1,094 (65.8)	835 (49.5)
Mobility^a^		
Completely dependent (*N*, %)	341 (20.5)	125 (8.4)
Mainly dependent (*N*, %)	230 (13.8)	149 (10.0)
Partially dependent (*N*, %)	304 (18.3)	249 (16.8)
Mainly independent (*N*, %)	399 (24.0)	243 (16.4)
Completely independent (*N*, %)	371 (22.3)	708 (47.6)
Dry skin overall (*N*, %, 95% CI)	684 (41.2, 38.8–43.5)	820 (55.2, 52.6–57.7)
Dry skin face (*N*, %)	274 (16.5)	417 (28.1)
Dry skin trunk (*N*, %)	325 (19.6)	421 (28.3)
Dry skin arms and hands (*N*, %)	380 (22.9)	616 (41.5)
Dry skin legs and feet (*N*, %)	631 (38.0)	724 (48.7)
Pruritus (*N*, %, 95% CI)^b^	163 (9.8, 8.5–11.3)	231 (15.5, 13.8–17.5)
Skincare dependent (*N*, %, 95% CI)^c^	1,428 (85.9, 84.2–87.5)	533 (35.9, 33.5–38.3)
Washed (*N*, %, 95% CI)^d^	1,415 (85.1, 83.3–86.8)	518 (34.9, 32.5–37.3)
Showered (*N*, %, 95% CI)^e^	1,200 (72.2, 70.0–74.3)	246 (16.6, 14.8–18.5)
Bathed (*N*, %, 95% CI)^f^	1,364 (82.1, 80.2–83.8)	493 (33.2, 30.8–35.6)
Leave‐on products applied (*N*, %, 95% CI)^g^	1,458 (87.7, 86.1–89.2)	476 (32.0, 27.7–34.4)
Face (*N*, %)	575 (34.6)	140 (9.4)
Trunk (*N*, %)	1,009 (60.7)	305 (20.5)
Arms and hands (*N*, %)	822 (49.5)	273 (18.4)
Legs and feet (*N*, %)	1,266 (76.2)	397 (26.7)

Note

Missing data nursing homes: a = 17, b = 87, c = 61, d = 174, e = 317, f = 28, g = 66. Missing data hospitals: a = 12, b = 140, c = 78, d = 810, e = 967, f = 134, g = 902.

### 3.2 Main results

In 2015 and 2016, the prevalence of dry skin was 41.2% (95% CI 38.8%–43.5%) in nursing homes and 55.2% (95% CI 52.6%–57.7%) in hospitals. Legs and feet were most often affected by dry skin, followed by arms and hands. About 16% of all hospital patients suffered from pruritus. In nursing homes, this applied to 10%.

Nursing home residents were on average more immobile compared with hospital patients (e.g., 20.5% vs. 8.4% completely dependent). The prevalence of skincare dependency was higher in nursing homes than in hospitals (85.9% [95% CI 84.2%–87.5%] versus 35.9% [95% CI 33.5%–38.3%]). Skincare activities were performed more often in nursing homes compared with hospitals. Regarding skin cleansing, most residents and patients were washed, followed by bathing. The proportions of patients being showered were lowest in hospitals (16.6% [95% CI 14.8%–18.5%]). Overall, 87.7% (95% CI 86.1%–89.2%) of nursing home residents received leave‐on products, and in hospitals, this applies to 32.0% (95% CI 27.7%–34.4%). The most frequent body parts treated with leave‐on products by caregivers were the legs and feet (76.2% in nursing homes and 26.7% in hospitals).

Table 2 presents the numbers and proportions of skincare‐dependent participants with dry skin. Dry skin overall was significantly higher in hospitals (70.2%) than in nursing homes (43.7%). It is also noticeable that in hospitals the proportion of patients with skin dryness was higher when being skincare dependent compared with the proportion in all hospital patients with dry skin (70.2% vs. 55.2% in Table 1). The body parts most often affected by skin dryness were “legs and feet” (40.5% in nursing homes, 65.9% in hospitals) and “arms and hands” (24.2% in nursing homes, 52.5% hospitals). In comparison with nursing homes, the proportions of hospital patients with moderate‐to‐severe forms of skin dryness were significantly higher regarding each considered body site.

**Table 2:** Skin dryness in skincare‐dependent residents and patients (2015 and 2016)

Skincare‐dependent participants with dry skin	Nursing homes (*N* = 1,428)	Hospitals (*N* = 533)	P[Link]
Dry skin overall (*N*, %)	624 (43.7)	374 (70.2)	<0.001
Dry skin face (*N*, %)	245 (17.2)	177 (33.2)	<0.001
Mild form (ODS 1)	176 (12.3)	129 (24.2)	<0.001
Moderate‐to‐severe form (ODS 2–4)	64 (4.5)	47 (8.8)	<0.001
Dry skin trunk (*N*, %)	291 (20.4)	205 (38.5)	<0.001
Mild form (ODS 1)	200 (14.0)	122 (22.9)	<0.001
Moderate‐to‐severe form (ODS 2–4)	86 (6.0)	82 (15.4)	<0.001
Dry skin arms and hands (*N*, %)	346 (24.2)	280 (52.5)	<0.001
Mild form (ODS 1)	227 (15.9)	147 (27.6)	<0.001
Moderate‐to‐severe form (ODS 2–4)	115 (8.1)	130 (24.4)	<0.001
Dry skin legs and feet (*N*, %)	578 (40.5)	351 (65.9)	<0.001
Mild form (ODS 1)	344 (24.1)	169 (31.7)	0.001
Moderate‐to‐severe form (ODS 2–4)	222 (15.5)	180 (33.8)	<0.001

Note

Missing data regarding severity of skin dryness: Nursing homes—face = 5, trunk = 5, arms and hands = 4, legs and feet = 12; Hospitals—face = 1, trunk = 1, arms and hands = 3, legs and feet = 2.

Chi‐square test.

Table 3 shows the numbers and proportions of skincare‐dependent nursing home residents who were receiving leave‐on products by nurses. Skincare‐dependent residents without dry skin, with mild form of skin dryness and with moderate‐to‐severe forms of skin dryness are presented separately. Residents without dry skin received most often leave‐on products at legs and feet (76.5%), followed by the trunk (59.6%), arms and hands (45.7%) and face (30.5%). In case of dry skin, the percentages of leave‐on product application increased clearly at each body area, with similar proportions in residents with mild form and more severe forms of skin dryness (e.g., 95.9% at legs and feet with mild skin dryness and 92.3% at feet and legs with moderate‐to‐severe skin dryness).

**Table 3:** Application of leave‐on products in skincare‐dependent nursing home residents (2015 and 2016)

Application of leave‐on products in skincare‐dependent residents	Without dry skin[Link] (ODS 0)	*N* = total (ODS 0)	With mild form[Link] (ODS 1)	*N* = total (ODS 1)	With moderate‐to‐severe form[Link] (ODS 2–4)	*N* = total (ODS 2–4)	P[Link]
Leave‐on product face (*N*, %)	361 (30.5)	1,183	132 (75.0)	176	53 (82.8)	64	<0.001
Leave‐on product trunk (*N*, %)	678 (59.6)	1,137	187 (93.5)	200	80 (93.0)	86	<0.001
Leave‐on product arms and hands (*N*, %)	494 (45.7)	1,082	194 (85.5)	227	92 (80.0)	115	<0.001
Leave‐on product legs and feet (*N*, %)	650 (76.5)	850	330 (95.9)	344	205 (92.3)	222	<0.001

Note

Missing data regarding severity of skin dryness: face = 5, trunk = 5, arms and hands = 4, legs and feet = 12.

Chi‐square test.

At corresponding body part.

In Table 4, the corresponding results are shown for hospital patients. The proportions of applied leave‐on products were lower at each body area compared with nursing homes. The body parts with the highest proportions of leave‐on product use in patients without dry skin were the trunk (34.5%) and legs and feet (32.4%). Like nursing homes, the proportions of leave‐on product applications per skin area were higher when patients had dry skin but remained lower than in nursing homes. Most treated body areas with dry skin were legs and feet (76.3% at legs and feet with mild skin dryness and 75.6% at legs and feet with moderate‐to‐severe skin dryness) and trunk (63.9% at trunk with mild skin dryness and 74.4% at trunk with moderate‐to‐severe skin dryness). Per skin area, the differences between proportions were statistically significant in both institutional types.

**Table 4:** Application of leave‐on products in skincare‐dependent hospital patients (2015 and 2016)

Application of leave‐on products in skincare‐dependent patients	Without dry skin[Link] (ODS 0)	*N* = total (ODS 0)	With mild form[Link] (ODS 1)	*N* = total (ODS 1)	With moderate‐to‐severe form[Link] (ODS 2–4)	*N* = total (ODS 2–4)	P[Link]
Leave‐on product face (*N*, %)	44 (12.4)	356	52 (40.3)	129	24 (51.1)	47	<0.001
Leave‐on product trunk (*N*, %)	113 (34.5)	328	78 (63.9)	122	61 (74.4)	82	<0.001
Leave‐on product arms and hands (*N*, %)	58 (22.9)	253	89 (60.5)	147	86 (66.2)	130	<0.001
Leave‐on product legs and feet (*N*, %)	59 (32.4)	182	129 (76.3)	169	136 (75.6)	180	<0.001

Note

Missing data regarding severity of skin dryness: face = 1, trunk = 1, arms and hands = 3, legs and feet = 2.

Chi‐square test.

At corresponding body part.

## 4 DISCUSSION

Results of these two multicenter prevalence studies indicate that approximately half of all nursing home residents and hospital patients were affected by dry skin. Results further indicate that these proportions are even higher in subjects who are skincare dependent. In other words, there seems to be an association between skincare dependency and dry skin, which has been proposed previously (Lichterfeld et al., 2016; Lichterfeld‐Kottner et al., 2018). The pattern of skin areas affected by skin dryness is also comparable to available evidence, which showed that dry skin was most prevalent at the distal extremities (Lichterfeld et al., 2016; Smith et al., 2002).

Study results further suggest that the application of leave‐on products seems to be an integral part of nursing practice in nursing homes, which is in accordance with previous research (Kottner, Rahn, et al., 2013; Rahn et al., 2016). Even without dry skin, about 75% of all skincare‐dependent residents received leave‐on products at legs and feet. Skincare‐dependent hospital patients received clearly fewer leave‐on products compared to nursing home residents. In both institutions, residents and patients were treated more often with leave‐on products when having dry skin. This indicates that the presence of signs of dry skin might trigger this skincare intervention. Nevertheless, the proportions of treated skin areas were still lower in hospitals compared with nursing home residents. This might indicate that less attention was given to dry skin in this setting.

There seems to be a stronger awareness of the dry skin condition in nursing homes. This might be explained by a stronger focus on “caring” in long‐term care and a stronger emphasis on “healing” in acute care. This assumption is supported by Osborne, Douglas, Reid, Jones, and Gardner (2015), who investigated the use of physical assessment skills in acute care nurses and midwives. The authors concluded that the nurses’ physical assessment mainly comprise vital signs, like measuring blood pressure, oxygen saturation or temperature. Though the inspection of the skin was performed regularly, this was rather focused on checking the colour, the presence of lesions or the inspection of wounds than on clinical signs of skin dryness. In another study, undiagnosed skin conditions in a Swiss internal medicine division were reported (Goeksu et al., 2012). The study physicians diagnosed xerosis cutis in 156 patients, of whom none had received treatment during the hospital stay and 76% stated that they had wished to receive treatment for this condition.

The high proportions of skincare‐dependent residents and patients who were affected by dry skin even though receiving skincare applications may question the effect of the respective skincare interventions. On one hand, there might be an undersupply, because residents and patients do not get the right products and the right quantity they need. On the other hand, inappropriate products may even aggravate signs of dry skin, especially when containing rather hydrophilic or irritating components. For adequate skincare, the choice of a leave‐on product should be based on the product's features like its lipophilic/hydrophilic property or the viscosity of the vehicle as well as on an individual assessment of the skin's condition and the extent of the affected body part (Surber & Kottner, 2017). An understanding of the range of emollient options (e.g., occlusive emollient cream, humectant‐containing emollient) is thus crucial for an appropriate decision‐making (Moncrieff et al., 2013). This requires an appropriate knowledge of practitioners and caregivers. The establishment of an evidence‐based guideline would be a main support and could further raise the awareness that skin dryness is not only a marginal aesthetic problem, but rather a health problem which requires appropriate skincare actions to prevent secondary skin diseases. Likewise, the well‐being of patients and residents can be increased by the application of leave‐on products due to, for example, the physical contact, the attention, a pleasant fragrance of the product and the sensation of smooth and flexible skin.

### 4.1 Limitations

Due to the voluntary participation of institutions, residents and patients, a selection bias might be present. The present study investigated how many people and which body parts were treated with leave‐on products by caregivers, but the suitability of the respective skincare intervention is unclear. Due to the cross‐sectional study design, no statements about causal relationships can be made. Regarding the assessment of skin dryness, no interrater reliability analysis was performed. Missing data, which were higher in hospital patients than in nursing homes residents regarding skincare activities, may be considered another limitation.

## 5 CONCLUSION

Hospital patients had a slightly higher prevalence of dry skin compared with nursing home residents. When only considering skincare‐dependent participants, the proportion of hospital patients with dry skin increased noticeably. The probability of being affected by dry skin in case of skincare dependency is nearly twice as high in hospitals compared with nursing homes.

The routine use of leave‐on products seems to play a major role in the daily nursing care practice in nursing homes but not in hospitals. In both institutions, the percentage of participants who received skincare products was higher when the dry skin was present. It seems that the application of leave‐on products is triggered by signs of skin dryness. However, considering the high amount of skin dryness in skincare‐dependent participants, interventions seem not to be successful. Results indicate a need for skincare improvement in future.

## CONFLICT OF INTEREST

The authors declare that they have no conflict of interests.
